# A first principles examination of phosphorescence

**DOI:** 10.1039/d2ra03447f

**Published:** 2022-09-07

**Authors:** Anjay Manian, Igor Lyskov, Robert A. Shaw, Salvy P. Russo

**Affiliations:** ARC Centre of Excellence in Exciton Science, School of Science, RMIT University Melbourne 3000 Australia anjay.manian3@rmit.edu.au salvy.russo@rmit.edu.au; Department of Chemistry, University of Sheffield Sheffield S3 7HF UK

## Abstract

This paper explores phosphorescence from a first principles standpoint, and examines the intricacies involved in calculating the spin-forbidden *T*_1_ → *S*_0_ transition dipole moment, to highlight that the mechanism is not as complicated to compute as it seems. Using gas phase acridine as a case study, we break down the formalism required to compute the phosphorescent spectra within both the Franck–Condon and Herzberg–Teller regimes by coupling the first triplet excited state up to the *S*_4_ and *T*_4_ states. Despite the first singlet excited state appearing as an *L*_b_ state and not of nπ* character, the second order corrected rate constant was found to be 0.402 s^−1^, comparing well with experimental phosphorescent lifetimes of acridine derivatives. In showing only certain states are required to accurately describe the matrix elements as well as how to find these states, our calculations suggest that the nπ* state only weakly couples to the *T*_1_ state. This suggest its importance hinges on its ability to quench fluorescence and exalt non-radiative mechanisms rather than its contribution to the transition dipole moment. A followup investigation into the *T*_1_ → *S*_0_ transition dipole moment's growth as a function of its coupling to other electronic states highlights that terms dominating the matrix element arise entirely from the inclusion of states with strong spin–orbit coupling terms. This means that while the expansion of the transition dipole moment can extend to include an infinite number of electronic states, only certain states need to be included.

## Abstract 2

Phosphorescence is naturally spin-forbidden, and weak. Consequentially, to build the operator, one needs help from other excited states. Unfortunately, sometimes some states are more difficult to deal with than others.

## Graphical abstract

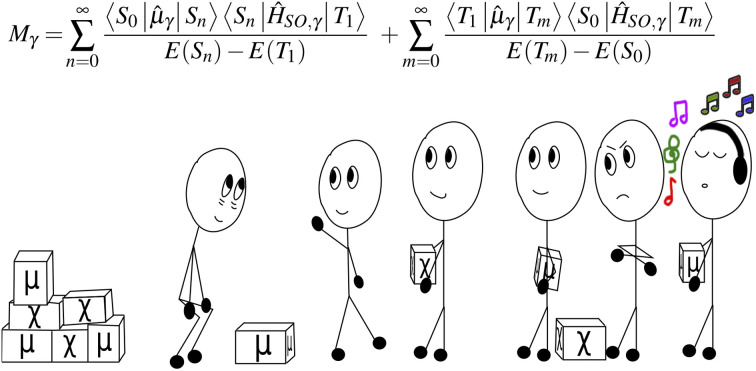

## 1 Introduction

Phosphorescence has the official definition of a radiative transition between states of different electronic spin multiplicities,^1–6^ but is more commonly understood as emission from first triplet excited state to the electronic ground state. While well known that photophysical properties of all compounds change with respect to temperature, systems prone to phosphorescence behave very differently.^3,4,7–11^ Interest in the photon harvesting applications of phosphorescent molecules has increased in the last few decades^12–21^ due to their recycling, or harvesting, of triplet excitons.

In addition to temperature, the phosphorescent phenomenon displays a sensitivity to molecular aggregation effects and the access of oxygen.^3–5,22–24^ Consequentially, most experimental studies are conducted under cryogenic and/or air-proof conditions, which seriously limits research into new more efficient molecular species. The reach of these results affects applications in many fields including but not limited to medical, photobiological, or optoelectronic fields. The most well known and efficient phosphorescent compounds are in the organometallic family of chromophores, such as metal sulfides or metal-cored porphyrins.^15,20,25–30^ However, fabrication of these kinds of molecules is neither cheap nor environmentally sound. The design of bio-friendly organic compounds for phosphorescent applications which are not limited by temperature or open-air is extremely difficult as the coupling between singlet and triplet excited states is typically quite weak. The mechanism therefore cannot compete with non-radiative processes such as internal conversion (spin is conserved) or inter-system crossing (spin is not conserved).^31,32^ While a myriad of well known biological compounds have been reported to phosphoresce,^33^ the rates are very slow. Consequentially, a method which could help predict the phosphorescence related properties of a candidate chromophore would facilitate those working to fabricate a more efficient species.

The photophysics of many heterocyclic and aromatic chromophores are defined by the spacing of two similarly characterised states, denoted as *L*_a_ and *L*_b_ states as per Platt's notation.^34^ The energetic ordering or spacing of these often degenerate states can change with respect to the medium, altering fluorescence and non-radiative decay pathways, a phenomenon known as solvatochromativity.^35,36^ Good examples for this include diketo-pyrrolopyrrole,^31,37,38^ indole,^39–41^ and acridine,^11,42^ with all three displaying drastically different photophysics depending on the polarity of the solvent it is measured in. Acridine in particular is interesting as in addition to the low lying *L*_a_ and *L*_b_ states, there is also a low lying nπ* state. In fact, this non-bonding state has been reported to appear as the emitting state in non-polar solvents and the gas phase.^11,42,43^

**Fig. 1:**
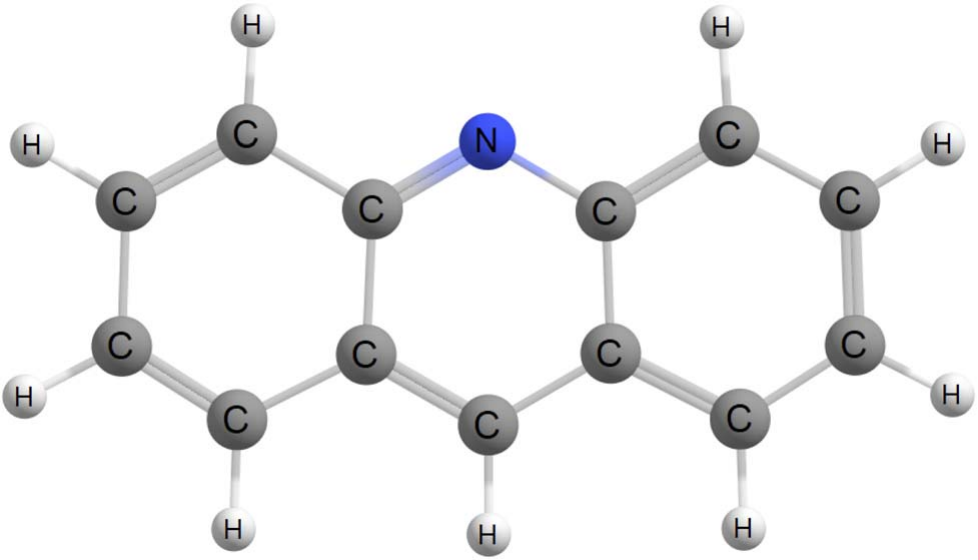
Schematic representation of the acridine chromophore.

Phosphorescence is a rare mechanism not only due to its impeding activation like temperature dependence and air-induced quenching due to oxygen contamination, but also because an exciton residing on a given excited state has to relax down to the first triplet excited state. In principle, it occurs for all compounds, but is literally outshined by fluorescence. However, if one considers the case where the fluorescing state is of nπ* character instead of ππ* character, fluorescence may be orders of magnitude smaller than the required inter-system crossing rate constant, allowing phosphorescence to compete. Further, acridine and it's derivatives has been reported to phosphoresce.^11,42–48^ For these reasons, we have chosen gas phase acridine, shown in Fig. 1, as a case study molecular system and will compute it's phosphorescence spectra and corresponding rate constant from first principles.

## 2 Theory

The rate of phosphorescence *k*_p_ can be calculated using Einstein's spontaneous emission function:^3,4,25,31,49,50^1
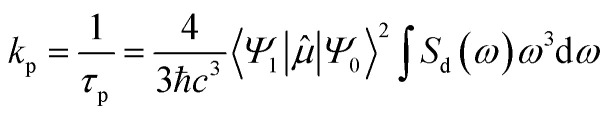
where *τ*_p_ is the phosphorescence lifetime, ℏ is Planck's reduced constant, *c* is the speed of light, 
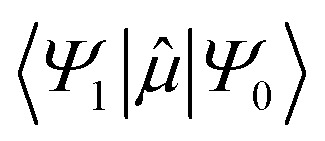
 is the transition dipole moment between the initial and final states of the transition, in this case describing the *T*_1_ → *S*_0_ transition, *S*_d_ is the normalised phosphorescence emission spectra, and *ω* is the energy. While computation of the transition dipole moment for same spin transitions is a standard option for most quantum chemical packages, the same cannot be said concerning 
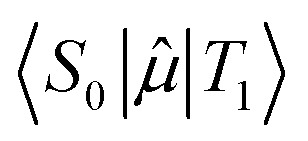
, which we will denote as *M* from this point onward for convenience. In order to bypass the forbidden nature of such a transition, we need to mix singlet and triplet state wavefunctions.^3,4,26,29,51,52^ Altering of electron spin is the result of strong spin–orbit coupling between states of different spin, so our transition dipole moment matrix elements have to be mixed with spin–orbit coupling matrix elements. If we choose to ignore vibronic effects and stay within the Franck–Condon (FC) regime,^53–55^ we can represent *M* as:2
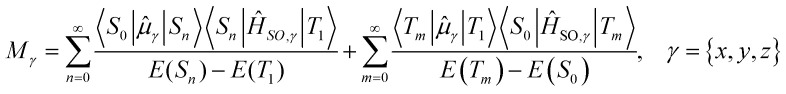
where *γ* is a given Cartesian coordinate, *n* denotes the singlet spin state, *m* denotes the triplet spin state, *Ĥ*_SO_ is the spin–orbit operator, and *E* is the energy of a given state. As the summation for both singlet and triplet manifolds goes to infinity, in theory every one should be included. However, as shown in the denominator of both terms, the contribution of a particular term approaches zero as the included state increases in energy. Here, we will opt to couple the first four excited states across both multiplicities to the *T*_1_ state.

We can then begin to consider the effects that each respective vibrational normal mode has to the rate constant, within the Herzberg–Teller (HT) regime.^56^ As explained by Lower & El-Sayed,^57^ singlet states of polyatomic molecules have some degree of triplet character, while triplet states have some degree of singlet character. Because this mixing is spin forbidden, our second order correction examines the derivatives with respect to the change in the spin–orbit coupling between states. To do this, we can expand the spin–orbit coupling operator at the Franck–Condon point *H*_SO_ with respect to the vibrational coordinates *Q*_*j*_ at the equilibrium geometry *Q*_0_ using a Taylor series, truncated at the first derivative term.^3,50^ This gives us:3
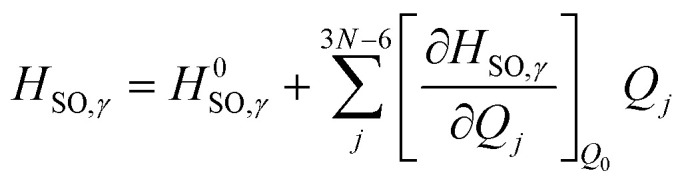


Here, the first term on the righthand side mediates the FC contribution while the second term mediates the HT contribution.

## 3 Computational details

Optimised††Unless otherwise stated, all calculation parameters, such as convergence criteria, are run using the default parameters listed in each respective software platform's manual. geometries were computed using a Complete Active Space Self Consistent Field (CASSCF) model with 6 electrons and 6 active orbitals, averaging across 5 roots using the Karlsruhe variant of the split valence with polarisation functions on non-hydrogen atoms def2-SV(P) basis set,^58,59^ as implemented in the MOLPRO software package.^60^ MOLPRO currently does not allow for solvent models with CASSCF calculations; as such we will calculate the properties of acridine in the gas phase. The electronic Hessian was then computed by finite differences using analytical energy gradients using the same CAS window and basis set. Corrections to the singlet point energies were then obtained by using the n-electron valence perturbation theory to the second order (NEVPT2) formalism^61–64^ as implemented in the ORCA software package,^65^ with the larger Karlsruhe triple zeta valence polarised def2-TZVP basis set.^66^

The singlet–singlet and triplet–triplet transition dipole moments were computed using the DFT based multireference configuration interaction DFT/MRCI method,^67,68^ using the def-SV(P) basis set. The one-particle basis was built using the Becke half-and-half Yang–Lee–Parr BHLYP exchange-correlation functional^69^ as implemented in the TURBOMOLE software package.^70^ The DFT/MRCI reference space was generated iteratively by including all electron configurations with expansion coefficients greater than 10^−3^ using 10 electrons across 10 orbitals for a maximum of two-electron excitations. Probe runs were calculated by discarding configurations with energy less than the highest reference energy; starting with a threshold of 0.6 *E*_h_, then 0.8, both within a paradigm of tight parameters,^68^ then using a threshold of 1.0 *E*_h_ using standard parameters. Spin–orbit matrix elements were computed using the SPOCK.CI module^71–73^ of the DFT/MRCI platform, within a quasi-degenerate perturbation theory framework. The spin–orbit coupling terms are computed using Breit-Pauli Hamiltonians,^73^ further simplified using a spin–orbit mean field approximation.^71^ Energies and coupling terms were calculated at the Franck–Condon point with respect to the initial state *i.e.* the 
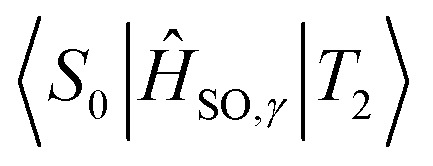
 spin–orbit matrix elements were computed at the *T*_2_ optimised geometry.

It should be noted that the triplet sublevels here are assumed to be degenerate as DFT/MRCI calculations are non-relativistic. As such, no zero-field splitting effects are included in this work.

The VIBES software package^74^ was used to compute the phosphorescence emission spectra, using 2^16^ integration points with a 300 fs time integral for integration, and a 100 cm^−1^ width for the Gaussian damping of the correlation function, all at a temperature of 300 K. For the calculation of derivative terms, numerical derivatives are calculated using a finite central difference method with a step size of *h* = 0.05 with respect to the mass-weighted normal coordinates. In order to account for the shifting of molecular orbital (MO) phase for each numerical derivative, a fictitious dot product was constructed using the configuration amplitudes of the leading configurations of the coupled states, and the largest MO coefficient of the underlying orbitals. Comparing these resulting products to the FC product clearly highlights when the phase has been reversed. For more details, see ref. 31 and 75.

## 4 Results & discussion

**Fig. 2:**
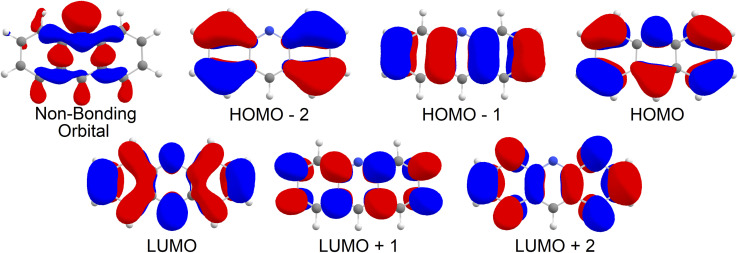
Molecular orbitals for acridine in the gas phase.

Upon analysis of the low lying excited states, which is summarised in Table 1, we note that none of our states are classified as an nπ* state. As shown in Fig. 2, the 6 orbitals included in the CAS window are all π type orbitals, and clearly does not include the non-bonding type highest occupied molecular orbital (HOMO). No missing orbitals were noticed within the lowest unoccupied molecular orbitals (LUMOs). Our results show that the first singlet excited state with an adiabatic energy of 3.19 eV is the *L*_b_ state as per Platt's notation, with mixed character of HOMO−1 → LUMO and HOMO → LUMO+1, while the second singlet excited state with an adiabatic energy of 3.92 eV is the *L*_a_ state, with a clear HOMO → LUMO character. Interestingly, the third singlet excited state with an adiabatic energy of 4.64 eV was found to be of double excitation quality as HOMO^2^ → LUMO^2^,‡‡The double excitation notation of HOMO^2^ is equivalent in form to HOMO, HOMO. while the fourth singlet excited state with an adiabatic energy of 4.84 eV was found to be mixed between HOMO^2^ → LUMO^2^ and HOMO → LUMO character. On the triplet manifold, the *T*_1_ state with an adiabatic energy of 1.59 eV was of *L*_a_ character, while the second excited state with an adiabatic energy of 3.59 eV was of *L*_b_ character. The third and fourth triplet excited states, with adiabatic energies of 4.41 eV and 4.67 eV respectively, were observed as mixed with HOMO−2 → LUMO and HOMO → LUMO+2 characteristics and of HOMO−1, HOMO → LUMO^2^ character respectively.

**Table 1:** Summary of adiabatic energies and orbital contributions for the lowest four singlet and triplet excited states at the FC point for each respective geometry. Energies are in units of eV

State	Energy	Orbital configuration	Contribution
1a^1^	—	GS	0.97
2a^1^	3.19	HOMO−1 → LUMO	0.32
		HOMO → LUMO+1	0.28
3a^1^	3.92	HOMO → LUMO	0.88
4a^1^	4.64	HOMO^2^ → LUMO^2^	0.65
5a^1^	4.84	HOMO^2^ → LUMO^2^	0.45
		HOMO → LUMO	0.17
1a^3^	1.59	HOMO → LUMO	0.88
2a^3^	3.59	HOMO−1 → LUMO	0.76
3a^3^	4.41	HOMO−2 → LUMO	0.39
		HOMO → LUMO+2	0.15
4a^3^	4.67	HOMO^2^ → LUMO^2^	0.51

Comparison of these results to work by Rubio-Pons and coworkers^43^ reports *L*_b_ and *L*_a_ singlet state energies of 3.28 eV and 3.34 eV respectively, both above an nπ* state with an energy of 3.18 eV. While initially this appears to be a cause for concern, closer observation shows the nπ* state to have a very small oscillator strength of 0.003, suggesting that the importance of this state is not in it's contribution to the strength of the *T*_1_ → *S*_0_ transition dipole moment, but rather in the quenching of fluorescence as to allow for non-radiative decay, evidence for which can be found in some spiro-compounds.^76^ Comparing our computed singlet energies to experimental work by Narva & McClure^77^ shows that while the energy of our *L*_b_ state may be reasonable, the *L*_a_ state is greatly overestimated. This is similarly seen for the triplet manifold as computed by Rubio-Pons and coworkers. While energies for the *L*_a_ triplet state published by Rubio-Pons and coworkers is within 0.2 eV of our calculated value, it is smaller by 0.4 eV as reported experimentally by Prochorow and coworkers.^44^ Conversely, the *L*_b_ triplet state is overestimated by 0.3 eV as reported experimentally by Kikuchi and coworkers.^45^ Further, it seems Rubio-Pons and coworkers also found an nπ* on the triplet manifold, appearing as the *T*_2_ state with an energy of 2.9 eV.

Investigation into why our results did not match with those reported by Rubio-Pons and coworkers shows that their calculation used a CAS window double the size of that used in this work (12,12), which is likely why the non-bonding orbital was not found within the low lying states. However, probe calculations run using the same window did not yield the expected non-bonding state either. There could be a number of reasons for this occurring, however this is currently outside the current scope of this work and will need to be reexamined in the future. Conversely for the experimental results, most phosphorescence measured for acridine is within crystalline matrices, which can be expected to have a strong effect on the photophysical properties of the compound. Juxtaposition of all these results shows a high degree of variability between data sets, and highlights the sensitivity of acridine and how strongly it can couple to its medium. Assuming the dielectric constant of 2,3-dimethyl naphthalene is similar to that of naphthalene^78^ at 2.87, and the dielectric constant of crystalline neon^79^ is 1.19, both experimental systems can be considered to reside in the low polarity scheme, and as such the *S*_1_ state can be expected to be of nπ* character. However results by Morawski & Prochorow show the *S*_1_ state in 2,3-dimethyl naphthalene to be of ππ* character, while Narva & McClure show it in biphenyl to be of nπ* type. From this discussion, one can see that despite differences in the ordering of excited states, the general photophysics match up well, likely due to the strong mixing between excited states of the acridine chromophore. As such, despite the lack of a near-degenerate nπ* state below the *L*_b_ state, we remain confident in the validity of these results, and their quantum chemical properties for use in our phosphorescent calculations.

When expanding *M*_*γ*_ across our included 8 states, *x*, *y*, and *z* Cartesian components of −1.98 × 10^−4^ au, −5.71 × 10^−8^ au, and −1.70 × 10^−5^ au were computed respectively, yielding a total moment of 1.99 × 10^−4^ au. These components are circled in red in Table 2. It is interesting to note that important contributions to each matrix element come from three states in particular: the fourth triplet excited state, and the second and third singlet excited states. Examination of the photophysical properties for each of these three states shows them to possess the only sizeable transition dipole moments or spin–orbit couplings to other states. In fact, the only large transition dipole components found were 0.71 au for the *x* component of the first singlet excited state, and 1.27 au for the *z* component of the second singlet excited state for singlet transition dipole moments, and −0.68 au for the *x* component of the second triplet excited state. Of all other components, only 5 others from a possible 18 were larger than 0.1 au. Spin–orbit coupling terms were in general much weaker, with only 2 components across a possible 27 being larger than 1 cm^−1^. Both these couplings were *x* components, for the second singlet and fourth triplet excited states, with couplings of −15.44 cm^−1^ and 19.05 cm^−1^ respectively.

**Table 2:** Evolution of *M*_*γ*_ as more singlet (*n*) and triplet (*m*) excited states are included in the expansion of the first order operator as per eqn (2). Elements in table are understood as *M*_*γ*_ when you terminate each summation at *n* and *m*. *μ*_*γ*_ and *E*_*m*,*n*_ are given in atomic units, while *H*_SO,*γ*_ is in cm^−1^. The parenthetic number shows the order of magnitude of the component *i.e.* 9.71(−9) ≡ 9.71 × 10^−9^. Blue circled elements show at which points *M*_*γ*_ receives a significant contribution, while Red circled elements show the element used for the rate calculation

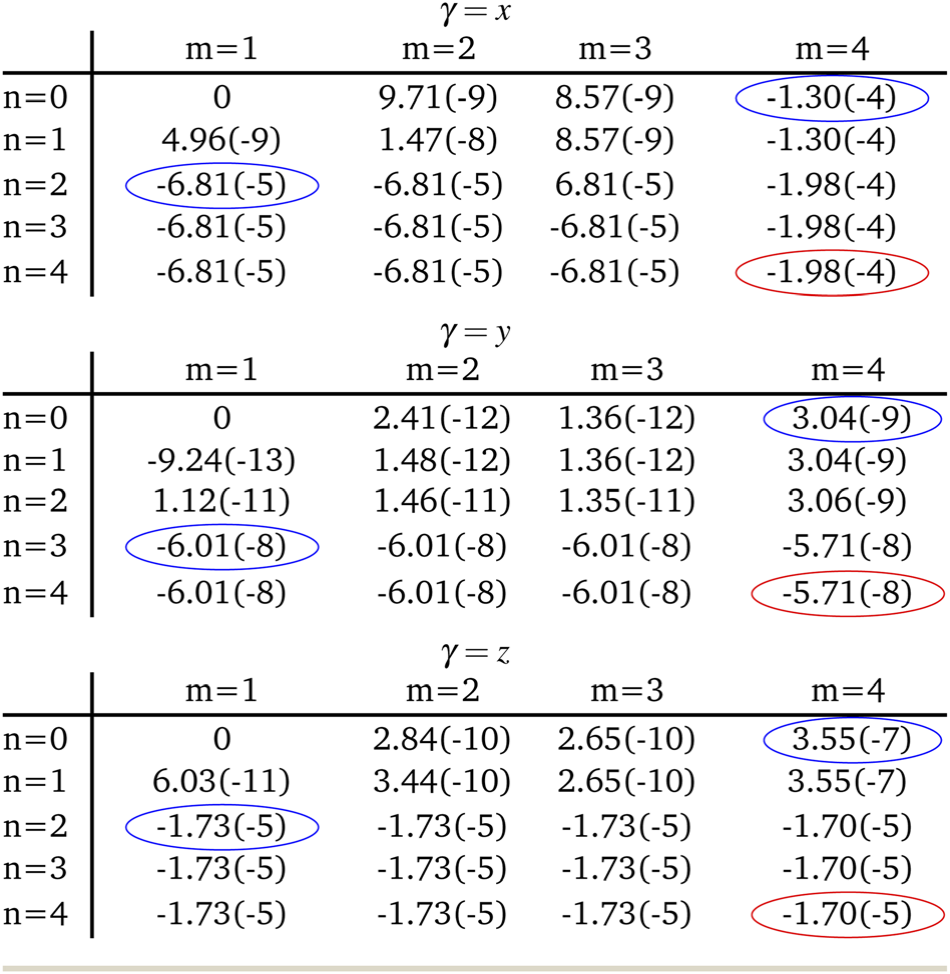

**Fig. 3:**
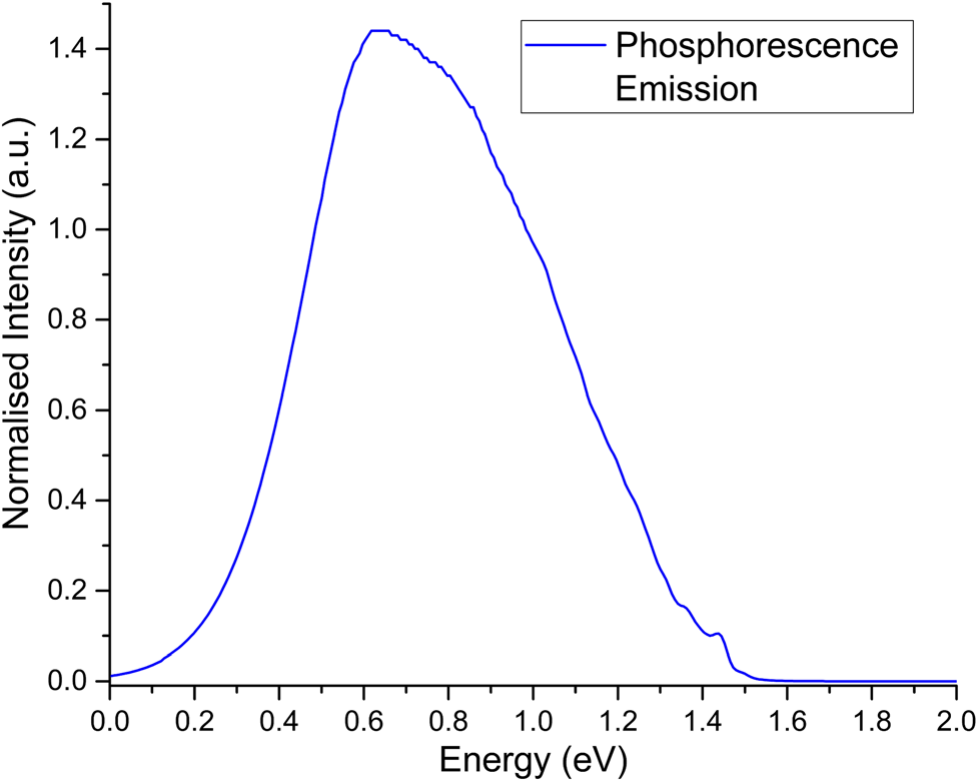
Second order corrected phosphorescence emission spectra predicted from first principles for acridine in the gas phase.

The second order corrected phosphorescent emission profile is shown in Fig. 3. The peak maximum can be found at around 0.65 eV, and extends all the way down to 1.5 eV before tapering off. At 1.4 eV, a very minor peak at the head of the bandshape can clearly be observed. Examination of the normal mode differences between electronic states shows three vibrational normal modes with Huang–Rhys factors larger than 0.5, indicative of very strong spectral features.^31,80^ Interestingly, the 10 normal modes with the largest Huang–Rhys factors are all between 400–2000 cm^−1^. Broadening over these peaks would certainly result in a strong bandshape, which is what is observed.

Spectra measured by Morawski & Prochorow^11^ shows two peaks measured within a dimethyl naphthalene crystalline matrix, as opposed to the single peak in this work. Considering the fact that they also note the phosphorescent spectra to be unexpectedly strong, it may be that the crystalline medium couples very strongly to acridine, which is why two peaks are observed instead of just one. A similar effect can be seen for acridine in 1 : 1 ethanol–methanol,^46^ and a neon matrix.^44^ The latter case is very interesting, as noble gas matrices have been used to emulate the gas phase such as interstellar conditions.^81,82^ However, while neon matrices and gas phase studies do yield very similar results,^83^ they are by definition different, such as how they treat vibrational properties.^84^ As such, any comparison between the two mediums should be treated with some scepticism.

**Fig. 4:**
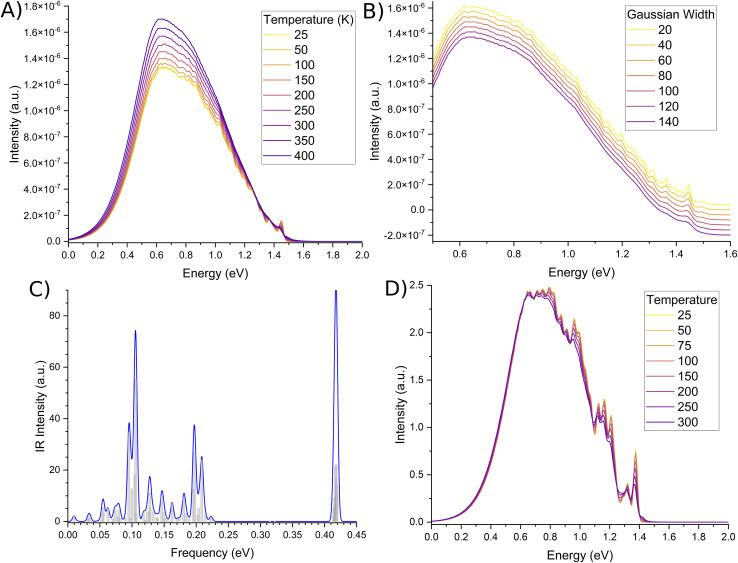
Fine-structure of the phosphorescence spectra. Second-order corrected spectra as a function of (A) temperature, and (B) Gaussian damping, in units of K and cm^−1^ respectively. Gaussian damping spectra have been offset to more clearly show the minute differences in the fine-structure. (C) Infra-red spectra of the *T*_1_ state, with a Gaussian broadening of 4.5 meV. (D) Phosphorescence due to only Franck–Condon terms are shown to illustrate possible convolution of modes resulting in an almost Gaussian lineshape. Spectra are not normalised.

While the phosphorescence bandshape is relatively smooth at room temperature, probing of the computational parameters elude to fine-structured bands. Temperature changes shown in Fig. 4A show the small peak at the head of the band to display a large degree of oscillation, similarly shown for small broadening functions as per Fig. 4B. These fine-structures are due to butterfly-type normal modes around 0.1 eV, breathing-line normal modes around 0.2 eV, and both of their high intensities, as per the infra-ref spectra in Fig. 4C. Conversely, oscillations along the spectral spine are due to deformations of the acridine core.

We also note that our emission peak is underestimated with respect to experiment. Based on the fine-structure of our spectra, we are confident this is due to some issue with the CASSCF optimised geometry. This becomes more clear when you examine the phosphorescence spectra calculated using different temperatures for only the Franck–Condon component, as per Fig. 4D. Here the spine is not-nearly as smooth as the second-order corrected lineshape. In fact, significantly more fine-structure details can be observed. In particular, it can be argued that based on the spectral details, there is at least one intense peak along the continuum that was convoluted and blurred. The CASSCF method was a necessity due to the number of high-lying excited states that needed to be computed. However, every derivative component is non-zero. As such, it may be that some vibrational components are being mis-estimated, leading to a more broadened spectral shape as opposed to two distinct peaks.

Application of eqn (1) within the Franck–Condon regime results in a rate constant of 0.0295 s^−1^ and a corresponding lifetime of 34 s. However, expansion of the matrix element within the Herzberg-Teller regime results in growth of *M* from 1.99 × 10^−4^ au to 7.79 × 10^−4^ au, and yields a second order corrected rate constant 0.402 s^−1^ and a corresponding lifetime of 2.5 s. This is an overall increase by a factor of ∼4 from the first-order to the second-order corrected transition dipole. Importantly, since eqn (1) is dependant on the square of *M*, the rate and lifetime are affected by a factor of ∼13. A perfect square relation is not observed due to a slight change in the spectra density as well as the transition dipole moment. While no experimental rate or lifetime could be found for the acridine core, this corrected lifetime does compare very well with the experimental rate for acridine orange of 0.56 s^−1^ as reported by Gryczyński and coworkers^47^ and reasonably well with the experimental rate for acridine yellow of 2.041 s^−1^ as reported by Fister & Harris.^48^

**Fig. 5:**
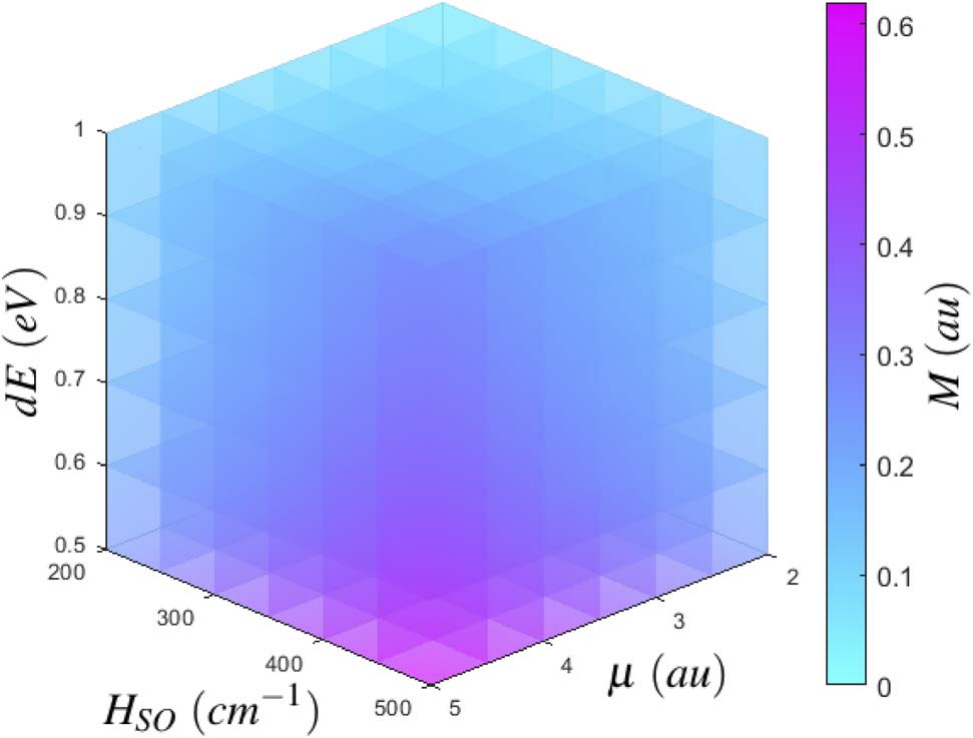
Scaling of *M* as a function of inter-state couplings. For an arbitrary state, *M* evolves by increasing either transition dipole matrix elements, spin–orbit coupling matrix element, or the energy separation between states.

If we consider the growth of *M*_*γ*_ as a function of it's dependent variables, we can begin to more clearly understand how to best maximise *M*_*γ*_. Eqn (2) has three major inputs: transition dipole components coupling *S*_*n*_ to the ground state or *T*_*m*_ to *T*_1_, spin–orbit coupling matrix elements between *T*_1_ and *S*_*n*_ or *T*_*m*_ to *S*_0_, and the energy gap between states of importance. If we consider an arbitrary state, we can plot the growth of *M*_*γ*_ with respect to increasing transition dipole components and inter-state energy.§§While this may be fairly self evident, we feel it is still worth visualising in order to clearly show how *M*_γ_ is affected by each of these three quantum chemical parameters. As per the density plot shown in Fig. 5, it becomes obvious that *M*_*γ*_ is maximised for larger transition dipole components with smaller energy gaps. While stronger quantum chemical coupling resulting in larger contributions to *M*_*γ*_ is obvious, it is surprising to see just how fast *M*_*γ*_ increases with respect to the spin–orbit coupling matrix element and inter-state energy. In fact, we see that *M*_*γ*_ is significantly more sensitive to spin–orbit coupling elements than it is to transition dipoles. Where the growth of *M*_*γ*_ with respect to *μ* could maximise at a value of 0.06 au for a state with a very strong transition dipole of 4–4.5 au, *M*_*γ*_ can gain even more strength with respect to an increasing *H*_SO_, whereby even a moderate spin–orbit component of 60 cm^−1^ can achieve a *T*_1_ → *S*_0_ transition dipole component of larger than 0.07 au. Should you have a chromophore with significant spin–orbit coupling, like organometallic compounds^85^ or large conjugated complexes,^86^ dominating contributions can be larger than 0.15 au for a spin–orbit matrix element of 200 cm^−1^.

In essence, we can therefore claim that while eqn (2) can be expanded to include an infinite number of electronic excited states, only certain states need to be included. Further, which states become important to *M*_*γ*_ could be probed from measurements at the ground state. Computing transition dipole moments for the lowest 30 states show that the 6^th^ singlet and 8^th^ triplet excited states possess large moments of 4.21 au and 2.60 au respectively. However, both of these states display poor spin–orbit coupling to any other state, and as shown in Fig. 5, strong spin–orbit coupling yields larger contributions to *M*_*γ*_, which is much more important than larger transition dipoles. In fact, the largest spin–orbit coupling component within the 30 lowest states not already included was the 
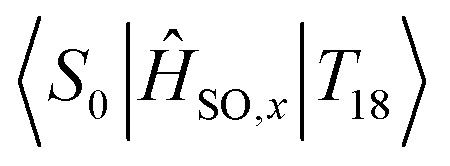
 component, with a coupling strength of 13.71 cm^−1^. However the energy separation would be much too large to yield a significant contribution to *M*_*γ*_, especially considering how small the spin–orbit coupling component is. As such, it is safe to conclude that we have included most of the important states in our description of phosphorescence in acridine.

With what was just shown, if we were to consider what is required to increase the rate of phosphorescence in any given way, we would be looking to increase the coupling between states. This means larger transition dipole components, larger spin–orbit matrix elements, and smaller energy gaps between electronic states. Larger transition dipole moments are difficult to engineer. Complexation is a common tactic which can drastically alter the photophysics of a compound, for the better with respect to the application. A good example of this is the difference between perylene and perylene diimide, where the former displays a reasonable oscillator strength^87^ which is increased drastically when compared to perylene diimide.^88^ One could also consider aggregation, where strong coupling in methanone dimers was shown to increase the phosphorescence lifetime.^24^ However, of the three quantum chemical properties, the easiest to alter is the strength of spin–orbit coupling. Heavy atoms^89,90^ or deuteration^91–94^ have been shown to increase spin–orbit matrix elements. Deuteration in particular has been known to affect the inter-system crossing pathway since the 1960's, with Hutchison & Mangum^91^ showing how deuteration changes spin-flipping rate constants in rigid gas naphthalene. Both Robinson & Frosch^92^ and Siebrand^93^ derived relations highlighting the effects of a change in reduced mass to the Franck–Condon factors and consequential changes in the density of states. However, Bixon & Jortner^95^ comment on how Siebrand missed the dominant contributions to the matrix element. This effect was demonstrated on azulene whereby an increase to the photoluminescence quantum yield of the second excited state was observed upon deuteration,^94^ implying a decrease to non-radiative processes. Design of solvatochromic compounds may also be worth considering, as a non-emissive *S*_1_ state would give the *T*_1_ state a greater chance to be occupied.

## 5 Conclusion

In this work, we have shown how one can begin from first principles and yield a phosphorescence rate constant, lifetime, and spectral profile. We used gas phase acridine as a case study in order to show how the transition dipole moment for a spin-forbidden transition could be calculated from readily available photophysical properties at the Franck–Condon point. We then discussed how to expand the operator to correct for Herzberg-Teller effects. Using our presented results, we were able to calculate a second order corrected rate constant which agreed very well with the experimental phosphorescent rate for acridine orange, despite the lack of a low-lying nπ* state. Further, we provided evidence that the nπ* state may have been weakly coupled to the first triplet excited state, resulting in a minimal contribution to the phosphorescence transition dipole moment and not necessary in the description of phosphorescent rate constants.

We were also able to show that while the operator can be expanded to include any number of electronic excited states, it is only those states which have either reasonably sized spin–orbit matrix element or very large transition dipole components that need to be included, the former being more important than the latter. In justification of this hierarchy of importance, we then explored what would be required to synthetically increase *M*_*γ*_, highlighting the importance and impact spin–orbit coupling has on the triplet to singlet transition dipole matrix element. From these results, and their discussions, we hope to have made phosphorescence transparent as a quantum chemical process, as well as providing some critical evidence of what is important to exalt the mechanism for photon harvesting applications.

## Conflicts of interest

The authors declare no conflicts of interest.

## Supplementary Material
